# Role of MDA5 in regulating CXCL10 expression induced by TLR3 signaling in human rheumatoid fibroblast-like synoviocytes

**DOI:** 10.1007/s11033-020-06069-z

**Published:** 2021-01-02

**Authors:** Tatsuro Saruga, Tadaatsu Imaizumi, Shogo Kawaguchi, Kazuhiko Seya, Tomoh Matsumiya, Eiji Sasaki, Norihiro Sasaki, Ryoko Uesato, Yasuyuki Ishibashi

**Affiliations:** 1 Department of Orthopaedic Surgery, Hirosaki University Graduate School of Medicine, 5 Zaifu-cho, Hirosaki, 036-8562 Japan; 2 Department of Vascular Biology, Hirosaki University Graduate School of Medicine, 5 Zaifu-cho, Hirosaki, 036-8562 Japan

**Keywords:** CXCL10, IFN-β, MDA5, Rheumatoid arthritis, TLR3

## Abstract

C-X-C motif chemokine 10 (CXCL10) is an inflammatory chemokine and a key molecule in the pathogenesis of rheumatoid arthritis (RA). Melanoma differentiation-associated gene 5 (MDA5) is an RNA helicase that plays a role in innate immune and inflammatory reactions. The details of the regulatory mechanisms of CXCL10 production and the precise role of MDA5 in RA synovitis have not been fully elucidated. The aim of this study was to examine the role of MDA5 in regulating CXCL10 expression in cultured human rheumatoid fibroblast-like synoviocytes (RFLS). RFLS was stimulated with Toll-like receptor 3 (TLR3) ligand polyinosinic:polycytidylic acid (poly I:C), a synthetic double-stranded RNA mimetic. Expression of interferon beta (*IFN-β*), *MDA5*, and *CXCL10* was measured by real-time quantitative reverse transcription polymerase chain reaction (qRT-PCR), western blotting, and enzyme-linked immunosorbent assay. A neutralizing antibody of IFN-β and siRNA-mediated MDA5 knockdown were used to determine the role of these molecules in regulating CXCL10 expression downstream of TLR3 signaling in RFLS. Poly I:C induced IFN-β, MDA5, and CXCL10 expression in a concentration- and time-dependent manner. IFN-β neutralizing antibody suppressed the expression of MDA5 and CXCL10, and knockdown of MDA5 decreased a part of CXCL10 expression (*p* < 0.001). The TLR3/IFN-β/CXCL10 axis may play a crucial role in the inflammatory responses in RA synovium, and MDA5 may be partially involved in this axis.

## Introduction

Rheumatoid arthritis (RA) is a common chronic inflammatory autoimmune disease that reduces quality of life [1]. The incidence of RA is considered to 0.5% to 1.0% in the world [2, 3]. Common characteristics of RA include infiltration of macrophages and lymphocytes, synovitis, and destruction of bone and cartilage [4]. An essential step in the pathogenesis of RA is the infiltration of the synovium by immune cells, which causes synovitis [5]. Thus, the fibroblast-like synoviocytes are critical cells in the pathogenesis of RA because these cells produce pro-inflammatory cytokines and proteases that contribute to cartilage destruction [6].

The innate immune system is the first line of protection against pathogenic microorganisms. The innate immune system is activated by pattern-recognition receptors that recognize pathogen-associated molecular patterns (PAMPs). In addition to PAMPs, damage-associated molecular patterns (DAMPs) released from dead and/or stressed cells can be also recognized by pattern-recognition receptors. DAMPs may induce sterile inflammation in the absence of infection and plays an important role in the pathogenesis of autoimmune diseases [7].

Toll-like receptors (TLRs) are the major pattern recognition receptors, and TLR3 is a receptor for double-stranded RNA (dsRNA). TLR3 is expressed in RA synoviocytes, and activation of TLR3 signaling induces the expression of various cytokines including vascular endothelial growth factor, interleukin-8 [8], C-C motif chemokine ligand 5 and C-X-C motif chemokine ligand 10 (CXCL10) [9]. The expression of TLR3 is reported to be higher than TLR2 and TLR4 in synovial fibroblasts [10]. Synovial inflammation may lead to the production of DAMPs in joints of patients with RA, and RNA released from necrotic synovial cells may activate TLR3 signaling in synoviocytes [11].

IFN-β, a type I IFN, is a key cytokine in innate immune reactions and exerts various functions by inducing the expression of hundreds of IFN-stimulated genes (ISGs). Melanoma differentiation-associated gene 5 (MDA5) is an ISG and was originally identified as a gene that was induced by IFN-β in melanoma cells [12]. MDA5 encodes a DExH box RNA helicase that functions as an ATPase [12], an inhibitor of melanoma growth [12], a cytosolic receptor for dsRNA generated during viral replication [13, 14], and a signaling molecule that regulates inflammatory reactions [15]. Although MDA5 is reported to be expressed in synovial fibroblasts [8], its precise role in RA synovitis has yet to be elucidated. Viral infections are known to exacerbate joint inflammation of RA [16]. In addition, higher levels of dsRNA were detected in synovial fluid of RA patients than osteoarthritis patients [17]. dsRNA is thought to be highly relevant to the pathogenesis of RA as DAMPs or PAMPs. Therefore, it is important to elucidate the role of TLR3 and MDA5.

CXCL10, also named interferon (IFN)-gamma-inducible protein-10, was initially identified as a chemokine secreted by several cell types including macrophages, endothelial cells, and fibroblasts in response to IFN-γ [9]. CXCL10 and IFN-γ mediate Th1 type immune reactions [18]. CXCL10 induces chemotaxis of CXC receptor 3 (CXCR3)+ leukocytes, including T cells and NK cells, and these leukocytes secrete cytokines that stimulate the expression of CXCL10 from other types of cells such as fibroblasts and endothelial cells. Thus, the CXCL10-CXCR3 axis regulates inflammatory responses and is involved in the pathogenesis of autoimmune diseases, such as RA and systemic lupus erythematosus [19]. However, details of the regulatory mechanisms of CXCL10 production in rheumatoid synoviocytes have not been fully elucidated.

The rationale for this study is that, in RA, the roles of TLR3 and MDA5 for synovitis and the regulation of CXCL10 are not well understood. We hypothesized that TLR3 and MDA5 are involved in the regulation of CXCL10 expression. The goal of this study was to investigate this hypothesis using cultured human rheumatoid fibroblast-like synoviocytes (RFLS).

## Materials and methods

### Reagents

Polyinosinic-polycytidylic acid (poly I:C; P1530) and rabbit anti-actin antibody were purchased from Sigma (St. Louis, MO). FBS was obtained from BIOWEST (Nuaille, France). Human type I interferon neutralizing antibody mixture (39000-1) was purchased from PBL Assay Science (Piscataway, NJ, USA). Small interfering RNA (siRNA) against MDA5 (SI03649037) and validated non-silencing negative control siRNA (1027281) were from Qiagen (Hilden, Germany). Lipofectamine RNAi-MAX reagent and M-MLV reverse transcriptase were from ThermoFisher (Carlsbad, CA, USA). The NucleoSpin RNA isolation kit was purchased from Macherey-Nagel (Duren, Germany). SsoAdvanced Universal SYBR Green Supermix was from Bio-Rad (Hercules, CA, USA). Primer oligo(dT)18 and oligonucleotide primers for polymerase chain reaction (PCR) were synthesized by Greiner Japan (Atsugi, Japan). A rabbit polyclonal antibody against MDA5 was from Immuno-Biological Laboratories (Fujioka, Japan). An enzyme-linked immunosorbent assay (ELISA) kit for CXCL10 was from R & D Systems (Minneapolis, MN, USA).

### Cells

Human RFLS cells from a patient were purchased from the Health Science Research Resources Bank (Sennan, Japan). This study has been approved by the Ethical Committee of Hirosaki University Graduate School of Medicine (2018-1117). The cells were cultured in Dulbecco’s modified Eagle medium (DMEM) supplemented with 10% fetal bovine serum. The cells were treated with 0.08–50 μg/mL poly I:C, and were incubated for up to 24 h. When we perform the experiments using the human type I IFN neutralizing antibody mixture, cells were pre-incubated for 1 h with the antibody mixture (1:25) and stimulated with 30 μg/mL poly I:C for 16 h. In order to observe the effect of MDA5 knockdown, cells were transfected with siRNA against MDA5 or non-silencing control siRNA using Lipofectamine RNAi-MAX reagent according to the supplier’s protocol. After a 48-h incubation, 30 μg/mL poly I:C was added to the cells.

### RNA extraction, reverse transcription and quantitative real-time PCR

RNA was extracted, and quantitative reverse transcription-PCR (qRT-PCR) was performed to measure *IFN-β*, *MDA5*, *CXCL10*, and glyceraldehyde-3-phosphate dehydrogenase (*GAPDH*) mRNA expression. Briefly, RNA was reverse transcribed using M-MLV reverse transcriptase and oligo(dT)18 primer. Amplification of cDNA was performed with gene-specific primers (Table 1) and SsoAdvanced Universal SYBR Green Supermix. *GAPDH* was used as an internal control. The data of *MDA5*/*GAPDH* were shown as fold increase compared with unstimulated cells. *IFN-β* and *CXCL10* mRNA were not detected in unstimulated cells, and the results of *IFN-β* and *CXCL10/GAPDH* were shown as arbitrary units.

**Table 1:** Oligonucleotide primers for quantitative real-time PCR

cDNA	Sequences
CXCL10-F	5′-TTCAAGGAGTACCTCTCTCTAG-3′
CXCL10-R	5′-CTGGATTCAGACATCTCTTCTC-3′
GAPDH-F	5′-GCACCGTCAAGGCTGAGAAC-3′
GAPDH-R	5′-ATGGTGGTGAAGACGCCAGT-3′
IFN-β-F	5′-CCTGTGGCAATTGAATGGGAGGC-3′
IFN-β-R	5′-CCAGGCACAGTGACTGTACTCCTT-3′
MDA5-F	5′-GTTGAAAAGGCTGGCTGAAAAC-3′
MDA5-R	5′-TCGATAACTCCTGAACCACTG-3′

### Western blotting

After the incubation, cells were washed and lysed with Laemmli sample buffer. Then, lysates were applied to polyacrylamide gel electrophoresis, and proteins were transferred onto polyvinylidene fluoride membranes. The membranes were blocked, and incubated with a rabbit antibody against MDA5 (1:2000) or actin (1:3000) for 16 h. Subsequently, the membranes were plobed with horseradish peroxidase (HRP)-conjugated anti-rabbit IgG antibody. Chemiluminescent for HRP substrate was used to detect the protein bands.

### ELISA

The cells conditioned medium were corrected, and determined using a commercially available ELISA kit according to the manufacturer’s protocol.

### Statistics

Data input and calculations were performed with SPSS ver. 12.0 J (SPSS Inc., Chicago, IL, USA). All experiments were performed at least three times. Values are reported as means ± standard deviation (SD). Differences between groups were analyzed using unpaired *t*-test. *P* value below 0.05 was considered statistically significant.

## Results

### Poly I:C induces the expression of IFN-β, MDA5, and CXCL10 in RFLS

**Fig. 1:**
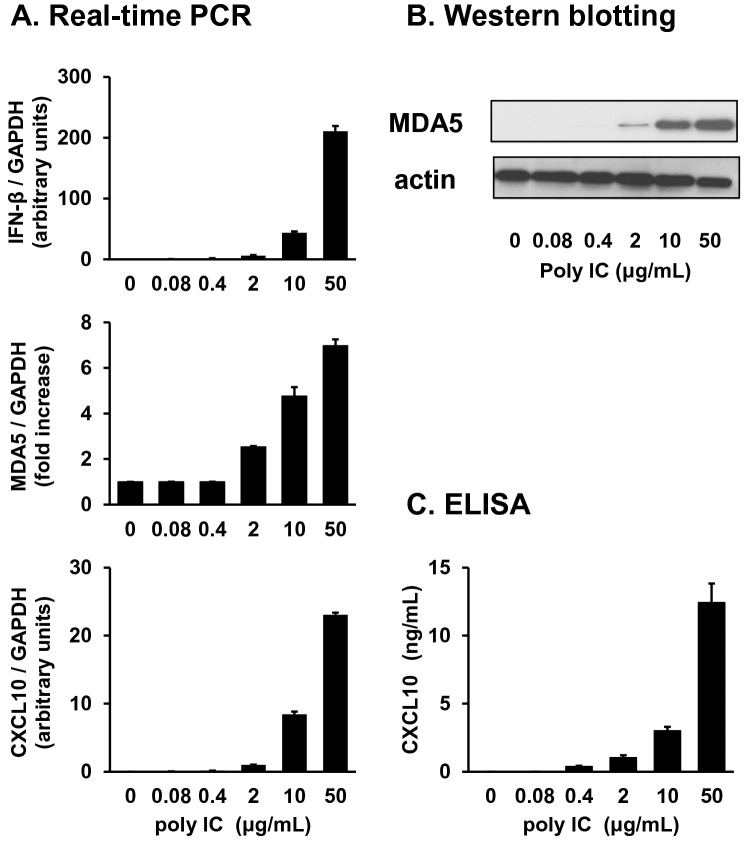
Treatment of human rheumatoid fibroblast-like synoviocytes (RFLS) with polyinosinic-polycytidylic acid (poly I:C) induces the expression of interferon beta (IFN-β), melanoma differentiation-associated gene 5 (MDA5), and CXCL10 in a concentration-dependent manner. RFLS were cultured and treated with 0.08–50 mg/mL poly I:C. (**a**) RNA was extracted after 4-h incubation. cDNA was synthesized and IFN-β, MDA5, CXCL10, or GAPDH cDNA was amplified using quantitative real-time PCR. Expression of IFN-β, MDA5, and CXCL10 mRNA was normalized with GAPDH. Data for MDA5 was shown as fold increase vs. unstimulated cells. IFN-β and CXCL10 mRNA expression was undetectable in unstimulated cells, and the fold increase for IFN-β and CXCL10 is shown as arbitrary units. (**b**) Cells were lysed after 16-h incubation, and lysates were subjected to western blot to detect MDA5 and actin protein expression. (**c**) Conditioned medium was collected after 24-h incubation, and the concentration of CXCL10 protein in the medium was measured using an ELISA kit. The data in (a) and (c) represent means ± standard deviation (*n* = 3)

**Fig. 2:**
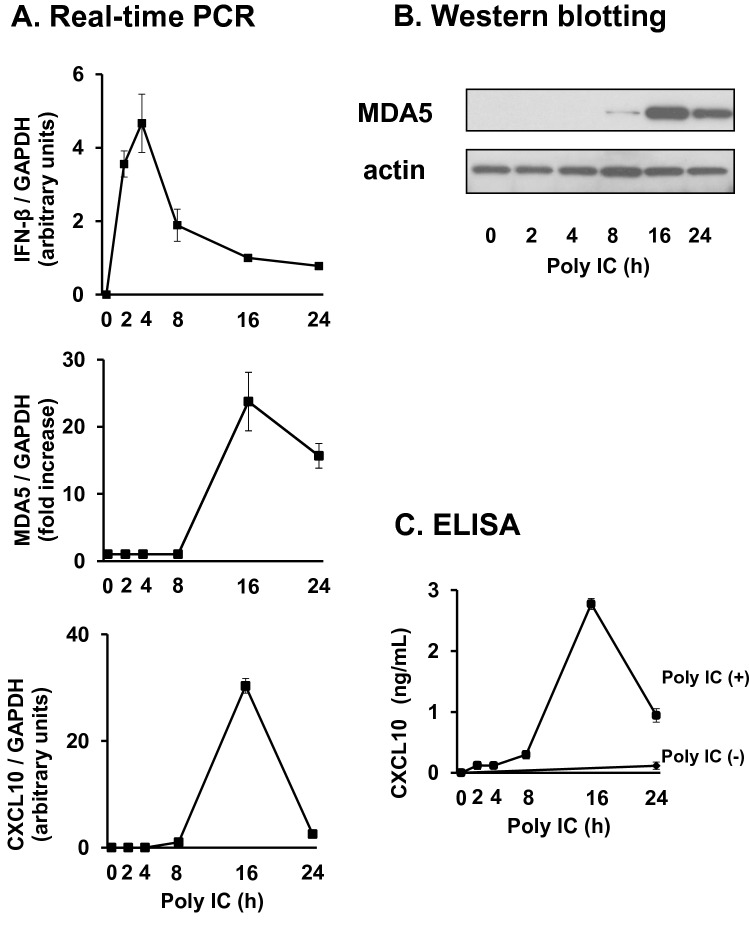
Poly I:C induces the expression of IFN-β, MDA5, and CXCL10 in a time-dependent manner. The cells were treated with 30 mg/mL poly I:C for up to 24 h. (**a**) RNA was extracted, and IFN-β, MDA5, CXCL10, and GAPDH expression was measured as in Fig. 1. (**b**) Cells were lysed, and western blotting for MDA5 and actin was performed. (**c**) Conditioned medium was collected and the concentration of CXCL10 protein was examined with ELISA. The data in (a) and (c) represent means ± standard deviation (*n* = 3)

Treatment of RFLS with poly I:C resulted in the induction of *IFN-β*, *MDA5*, and *CXCL10* in a concentration- (Fig. 1) and time-dependent manner (Fig. 2). By stimulation with poly I:C, *IFN-β* mRNA expression increased rapidly, reached its highest level after 4 h, and quickly decreased (Fig. 2a, upper panel). *MDA5* mRNA expression increased after *IFN-β* and peaked at 16 h (Fig. 2a, middle panel). *CXCL10* expression also reached its maximum at 16 h (Fig. 2a, lower panel). MDA5 (Fig. 2b) and CXCL10 (Fig. 2c) protein expression was also highest at 16 h.

### IFN-β mediates poly I:C-induced expression of MDA5 and CXCL10

**Fig. 3:**
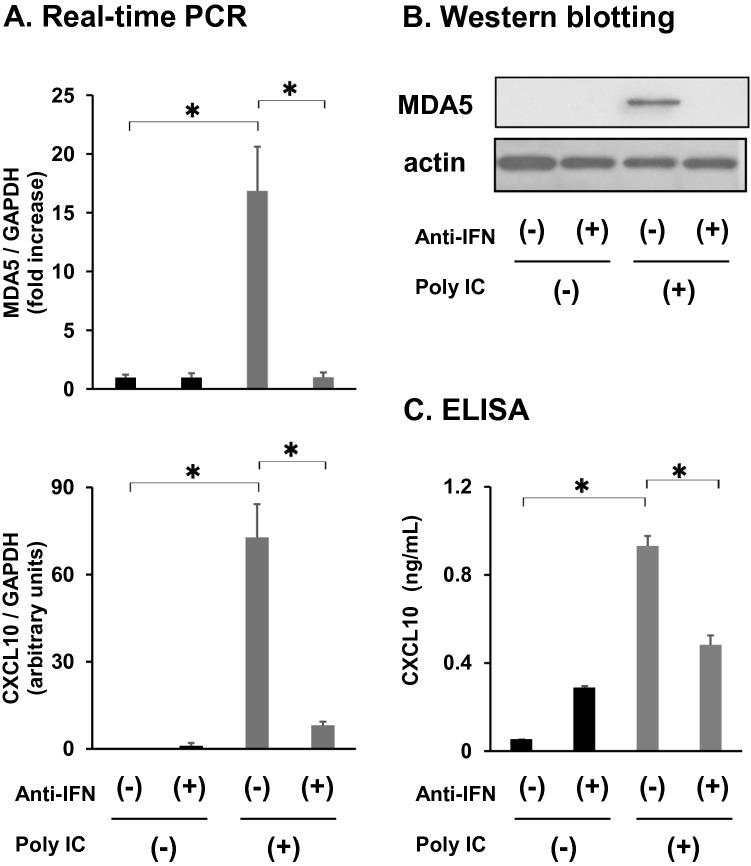
Neutralization of IFN-β decreases MDA5 and CXCL10 expression induced by poly I:C. Cells were pre-incubated for 1 h with human type I IFN neutralizing antibody mixture (1:25 dilution) before being treated with 30 mg/mL poly I:C for an additional 16 h. (**a**) RNA was extracted, and qRT-PCR was performed. (**b**) Cells were lysed, and MDA5 and actin expression was determined with western blot. (**c**) Medium was collected and subjected to CXCL10 ELISA. The data in (a) and (c) represent means ± standard deviation (*n* = 3). *p < 0.01, by *t*-test

Pretreatment of cells with neutralizing anti-IFN antibody mixture significantly decreased the induction of *MDA5* and *CXCL10* mRNA (Fig. 3a). MDA5 (Fig. 3b) and CXCL10 protein (Fig. 3c) expression was also inhibited by the anti-IFN antibody mixture.

### MDA5 is involved in poly I:C-induced CXCL10 expression

**Fig. 4:**
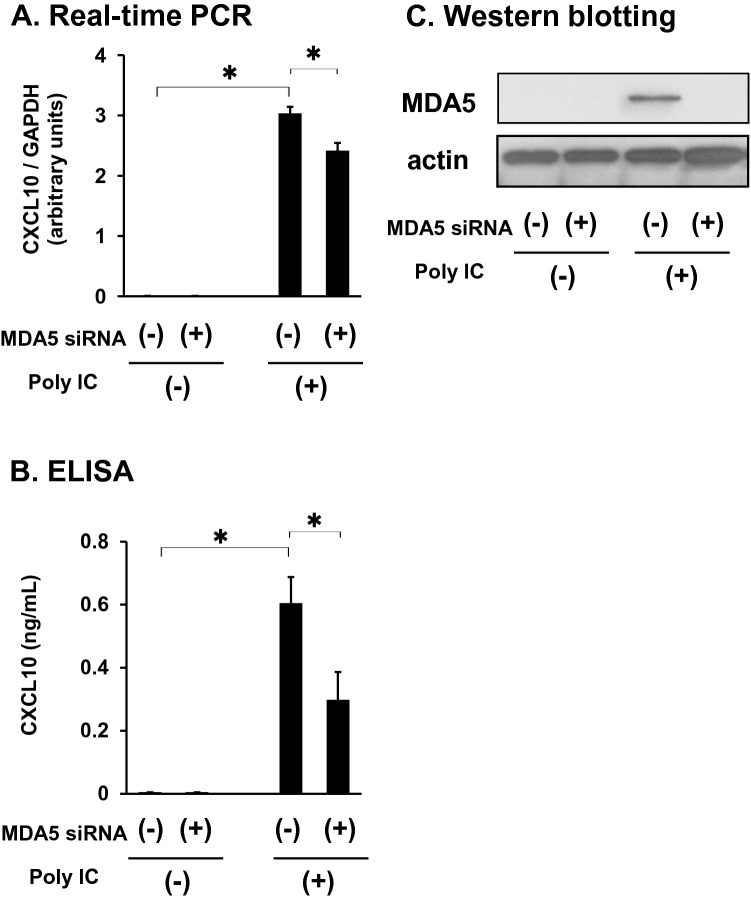
MDA5 knockdown decreases CXCL10 expression induced by poly I:C. Cells were transfected with a specific small interfering RNA (siRNA) against MDA5 and incubated for 48 h. Then, cells were treated with 30 mg/mL poly I:C for an additional 16 h. (**a**) RNA was extracted, and mRNA expression was examined using qRT-PCR. (**b**) Medium was collected and subjected to CXCL10 ELISA. (**c**) Cells were lysed and subjected to western blot as in Figure 1. Data in (a) and (b) represent means ± standard deviation (*n* = 3). *p < 0.01, by *t*-test

siRNA-mediated knockdown of *MDA5* significantly diminished *CXCL10* mRNA (Fig. 4a) and protein (Fig. 4b) expression induced by poly I:C. MDA5 knockdown was confirmed with western blot (Fig. 4c).

## Discussion

**Fig. 5:**
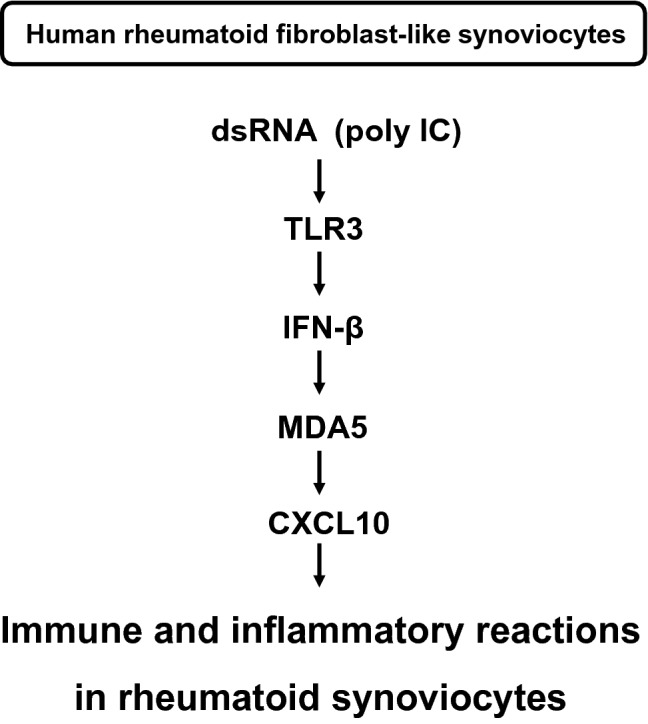
Proposed signaling pathway of TLR3-mediated CXCL10 expression in human rheumatoid fibroblast-like synoviocytes

We have demonstrated that the activation of TLR3 signaling in RFLS upregulated IFN-β, MDA5, and CXCL10 expression. In addition, neutralization of IFN-β inhibited MDA5 and CXCL10 expression, and knockdown of MDA5 decreased a part of expression of CXCL10. This suggests that TLR3 signaling induces IFN-β expression, and newly synthesized IFN-β mediates the expression of CXCL10 at least in part via MDA5 in RFLS. Taken together, the TLR3/IFN-β/CXCL10 axis may be involved in the inflammatory response of rheumatoid synovitis, and may be partially mediated by MDA5 (Fig. 5).

CXCL10 protein expression is elevated in serum [20] and synovial fluid [21] from patients with RA. In addition, serum CXCL10 expression is associated with better response to abatacept treatment [22]. Inhibition of the CXCL10/CXCR3 axis has been shown to downregulate the infiltration of inflammatory cells, including macrophages and T cells, into involved joints and to reduce the severity of synovitis and bone and cartilage destruction in animal models of RA [23]. CXCL10 also increases RANKL (receptor activator of NF-kB ligand) expression in CD4+ T cells [23]. Human phase II clinical trials using anti-CXCL10 monoclonal antibody (MDX-1100) in RA patients reported an improvement in response rate [24]. These findings suggest that CXCL10 plays a crucial role in the pathogenesis of RA, and it is important to clarify the mechanisms by which CXCL10 expression is regulated in RFLS.

The expression of CXCL10 in RFLS is augmented by various stimuli including inflammatory cytokines tumor necrosis factor alpha and IFN-γ [25] and TLR3 agonist poly I:C 11]. However, the regulatory mechanisms of CXCL10 production in RFLS have not been fully characterized. The TLR3/IFN-β/MDA5/CXCL10 axis demonstrated by our study corroborates previous findings of the role of MDA5 in regulating CXCL10 expression in glomerular mesangial cells [15], brain microvascular endothelial cells [26], and hepatocellular carcinoma cells [27]. The present study revealed a new role of MDA5 in RA synoviocytes and offers new insights about RA sterile inflammation mechanisms. However, the detailed molecular mechanisms by which MDA5 regulates CXCL10 expression should be further investigated.

The results of the present study suggest that IFN-β may positively contribute to synovial inflammation downstream of TLR3 signaling. However, this seems to be in contradiction to a previous study which showed that IFN-β has a protective effect against joint destruction [28]. IFN-β induces the expression of various kinds of ISGs, and ISGs include both pro-inflammatory and anti-inflammatory molecules. We hypothesize that MDA5 and CXCL10 are among the pro-inflammatory ISGs stimulated. The balance of pro-inflammatory and anti-inflammatory ISGs may be important for regulating inflammation in synoviocytes.

It is known that TLR3 functions as a receptor for poly I:C when poly I:C was just added to the culture medium of cells [15, 21, 23]. On the other hand, MDA5 can be a cytosolic RNA receptor when the cells were transfected with poly I:C complexed with cationic-lipid. In the present study, we added poly I:C alone to cells, and it is likely that poly I:C induced down-stream signaling via TLR3. However, we have not excluded the possibility that poly I:C bound to MDA5. This should be investigated in future studies. The knockdown of MDA5 only partially suppressed the expression of CXCL10. Our results suggest that MDA5 is at least partially involved in CXCL10 expression. The extent to how MDA5 is involved in CXCL10 expression is not clarified in this study, and further studies are required. Because this study was performed using only in vitro culture system, the observation of this study should be confirmed using in vivo model in future study. We showed the possible involvement of MDA5 in CXCL10 expression by dsRNA. CXCL10 produced by intra-articular dsRNA may enhance leukocyte infiltration and exacerbate RA inflammation. If the involvement of MDA5 in RA inflammation is further elucidated, MDA5 would be a new attractive therapeutic target for RA.

## Conclusions

We conclude that activation of TLR3 signaling induces the expression of IFN-β, MDA5 and CXCL10 in cultured human RFLS. IFN-β mediates MDA5 expression which, at least in part, regulates CXCL10 expression. The TLR3/IFN-β/CXCL10 axis may play a crucial role in the inflammatory responses in RA synovium, and MDA5 may be partially involved in this axis.

## Data Availability

The datasets used and analyzed in the current study are available from the corresponding author on reasonable request.
